# The impact of multiple long-term conditions on engaging with and maintaining behaviour change in older people with mild frailty: A qualitative study

**DOI:** 10.1177/26335565261448335

**Published:** 2026-04-29

**Authors:** Tasmin Alanna Rookes, Jessica Catchpole, Kate Walters, Yolanda Barrado-Martín, Sarah Kalwarowsky, Christina Avgerinou, Benjamin Gardner, Rebecca L. Gould, Paul Chadwick, Jane Hopkins, Vari M. Drennan, Kalpa Kharicha, Louise Marston, Claire Jowett, Rashmi Kumar, Rekha Elaswarapu, Rachael Frost

**Affiliations:** 1 Research Department of Primary Care and Population Health, 4919 University College London, London, UK; 2 Department of Experimental Psychology, 4919 University College London, London, UK; 3 School of Psychology, 3660 University of Surrey, Guildford, UK; 4 Division of Psychiatry, 4919 University College London, London, UK; 5 Division of Psychology & Language Sciences, 4919 University College London, London, UK; 6 Patient and Public Involvement Contributor, London, UK; 7 Centre for Health and Social Care Research, 4264 Kingston University, London, UK; 8 Health and Social Care Workforce Research Unit, The Policy Institute, 4616 King’s College London, London, UK

**Keywords:** behaviour change, self-management, frailty, multiple long-term conditions, qualitative interviews

## Abstract

Managing multiple long-term conditions (MLTCs) is a growing priority for health and social care systems, as MLTCs often lead to frailty and reduced resilience to adverse health events. Behaviour change interventions for this population have shown limited effectiveness. We developed HomeHealth, a home-based behaviour change intervention for older adults with mild frailty and evaluated it in a randomised controlled trial in England. As part of the process evaluation, we conducted a qualitative sub-study to examine barriers and facilitators to engagement, approaches to goal setting, and strategies for tailoring future interventions. Forty-nine participants with MLTCs who received HomeHealth were interviewed, within 6-months of their final intervention session. Participants had an average age of 80.3 years, 65% female, 76% white British, and lived with an average of 5.1 health conditions (range 2–11). Data were thematically analysed. Three themes were developed: (1) prioritising symptoms over conditions; (2) coping with and adapting to symptoms; and (3) tailoring goal setting for MLTCs. Impacts were driven by cumulative symptom burden rather than diagnoses, with mobility-related impairment being the primary concern. Symptom-focused goal setting supported engagement, but symptom fluctuations hindered progress. Findings underscore the importance of person-centred approaches. Targeting goals around functional impairment and symptom management may improve engagement compared to condition-focused strategies. Supporting adaptive behaviours during symptom exacerbations and providing positive feedback on effort, rather than completion, could sustain motivation and promote long-term behaviour change.

## Introduction

Multimorbidity is the presence of Multiple Long-Term Conditions (MLTCs) and is defined by the co-occurrence of two or more chronic conditions in the same individual. It is estimated that 37% of the global population is affected by MLTCs, with greater prevalence (67%) among those 74 years and over.^1,2^ MLTCs are often associated with disability, decline in functioning levels and quality of life, and higher healthcare costs.^3^ Within an ageing population context where both the proportion of older people and overall life expectancy is rising, managing MLTCs is a growing priority for health and social care systems.^4^

Frailty, a syndrome where people struggle to recover from adverse health events, is often present in older adults alongside Long-Term Conditions (LTCs).^5^ Whilst most older people who are frail are also living with MLTCs, fewer of those living with MLTCs are also frail.^6^ Living with MLTCs can contribute to cumulative deficits that can lead to frailty. Despite the high prevalence of MLTCs, evidence on the effective management of these conditions in the context of frailty is still scarce.^7^

MLTCs represent a significant challenge to healthcare services, policymakers, and researchers, as issues around patient complexity, morbidity burden and the interrelationships between MLTCs are poorly understood.^8^ Current care is organised around individual conditions and discrete specialties, which do not respond to the clinical challenges of MLTCs, such as competing healthcare needs and complex decision making, and is cost-ineffective.^8,9^ There are research, clinical, and policy imperatives to develop the evidence base and understanding around the unique and intersecting characteristics of MLTCs, and their effective integrated management. Likewise, there is a growing case for holistic, responsive, person-centred care, where quality of life and improved functioning are prioritised over disease-led treatment, characterised by multiple, potentially competing, therapies and polypharmacy.^10,11^ This is reflected in organisation of MLTC healthcare being a priority of the James Lind Alliance.^12^

So far, interventions delivered to people living with MLTCs, to improve medication adherence, self-management, and health promotion, have struggled to demonstrate their added value,^13–16^ beyond improving patients’ experiences.^17^ Tailored, behaviour change interventions beyond the healthcare domain could lessen the impact of MLTCs by improving health-related quality of life and decreasing symptoms of depression.^18^ Behaviour change interventions contain sets of techniques which are designed to change an individual’s behaviour patterns.^19^ People living with MLTCs may be faced with specific difficulties, such as accessibility and experiencing pain or fatigue, from the confluence of different health conditions that could interfere with their adherence to, and obtaining potential benefits from, these interventions.^20^ Hence, there is a need to understand the extent to which their conditions impact their everyday life and activities, participation in behaviour change, how different conditions interact, and what strategies seem successful among those living with MLTCs.

The present study was embedded in a single-blind, two arm randomised controlled trial assessing the effectiveness of the HomeHealth intervention (a tailored, behaviour change intervention, see Box 1) in people over 65 living with mild frailty (ISRCTN54268283). The intervention aimed to help older adults with mild frailty maintain their independence through asset-based goal setting, action planning, and problem solving to prevent further decline. A parallel mixed methods process evaluation, conducted alongside the main trial and reported elsewhere,^21,22^ assessed the fidelity, reach, and dose of the HomeHealth intervention, and analysed the acceptability, mechanisms, and impact of the intervention.

### Aims

1. To explore how MLTCs impact people’s approaches to behaviour change, such as setting and achieving goals.
2. To explore the barriers and facilitators to engagement with and benefits from the HomeHealth service for older people with mild frailty in those living with MLTCs.
3. To explore potential ways of tailoring interventions relevant for such a diverse patient group.

## Methods

We carried out a pre-planned, separately funded, qualitative sub-study embedded within a mixed methods process evaluation, including data from intervention participants.

**Box 1. The HomeHealth service^23^**

HomeHealth is a person-centred, asset-based behaviour change intervention that aimed to promote independence and prevent decline in people aged 65 and over, living with mild frailty (as defined by the Clinical Frailty Scale^24^). The intervention was developed through literature reviews, qualitative interviews, and co-design workshops, to ensure it was meeting the needs of people with mild frailty. Three hundred and eighty-eight community-dwelling people were recruited over three sites in urban and semi-rural areas (North London, Bradford, and Hertfordshire), 195 of whom were allocated to the intervention group and offered tailored support in their home. Most appointments were face-to-face, with a small number of remote appointments. Seven support workers were recruited from non-clinical health and voluntary sector backgrounds and received training in behaviour change, as well as communication skills, strength and balance exercises, psychological wellbeing, and nutrition. They visited participants monthly over six months, to understand what was important to the participant. Drawing on the ‘COM-B’ model of behaviour change (Capability, Opportunity, Motivation – Behaviour),^19^ support workers helped participants set outcome goals around improving or maintaining mobility, cognition, psychological wellbeing, social interactions, nutrition, or other areas relevant for the participant. Outcome goals were subdivided into behaviour goals (specific actions the participant could take to achieve the outcome goal) and SMART goals (specific, measurable, achievable, relevant, and timely action plans to achieve the behaviours). Goals were reviewed at each appointment.

### Participant interviews

As part of the HomeHealth process evaluation, led by YBM, we conducted semi-structured qualitative interviews with participants between 1- and 6-months from them receiving their final intervention session. We purposively sampled to ensure diversity in age, ethnicity, sex, deprivation level, educational level, number of sessions completed, support worker, study site, and goal type. To determine whether participants had more than one LTC, medical notes were extracted from GP records, and this information was supplemented by self-reported LTCs described and identified by participants as part of the interview. Eighty-six older people were invited to interview, with an information sheet, reply letter, and freepost envelope sent via post, when they had completed 6-month follow-up and were no longer receiving intervention sessions. In total, 48 of these were interviewed, 16 declined, 17 did not respond and five expressed an interest to be interviewed but then withdrew. One additional older person contacted the research team and initiated an interview. All participants self-reported having more than one long term condition during interviews. This resulted in all 49 interviewed participants being included in this sub-study analysis.

JC (n=19), TAR (n=16), SG (n=11), YBM (n=2), and RF (n=1) conducted the interviews, following the same topic guide, with older people between August 2022 and May 2023. All researchers self-identify as female presenting with an interest in ageing. Weekly team meetings and debriefs, reflecting on the project aims and interview data, ensured all team members were aligned with their approach. Although, it is likely that individual differences played a role in probing and prompting within the interviews, which are at the centre of reflexive thematic analysis. Participants were given the choice of meeting face-to-face in their homes, by telephone, or videoconferencing. Most (n=43, 88%) participants chose face-to-face interviews, with two videoconferencing and four by telephone. Participants were given the option to have a friend, carer, or partner present, but only three chose this, although on some interviews, a participant’s partner intermittently participated in the interview and added to the dialogue, which was included in the analysis. Interviews lasted on average 69 minutes (range 35–124 minutes). Participants were given a £20 voucher for their participation.

The topic guide for the semi-structured interviews was developed in line with the evidence gaps identified in the systematic reviews conducted as part of developing the intervention and to answer the research aims of this work.^25–27^ The process evaluation topic guide, including topics related to MLTCs, was initially developed by YBM, with support from RF, KW, and public contributors during the first 6-months of the intervention being delivered within the trial. It was then iteratively updated during weekly process evaluation team meetings to facilitate conversation flow, and further refined with wider team feedback, including public contributors. Topics relating to MLTCs included physical and mental health conditions, symptoms, medications, impact on everyday life and impact upon health-promoting behaviours and engagement with goal setting and maintenance. Broader topics included the health impacts of COVID-19, memory difficulties, acceptability of HomeHealth, interactions with the support worker and other facilitators and barriers to goal setting. Researchers kept reflective diaries after each visit and shared reflections with the other researchers during weekly team meetings. Interviews were audio-recorded and transcribed verbatim by a third-party service, checked for accuracy, and then anonymised for analysis.

### Data analysis

Codebook thematic analysis^28^ was initially used to analyse data. After independently reading transcripts and feedback from co-authors, TAR and YBM created an inductive initial coding framework, with feedback from the wider team to which new codes were added as coding progressed, using NVivo 12. TAR, JC, SG, YBM, and RF coded the transcripts using this framework. Comprehensive coding on all topics was applied to all interviews, with data from relevant codes used for this sub-study.

This sub-study analysis was led by JC and TAR. For the data relevant to this sub-study, reflexive thematic analysis^28^ was pursued and supplemented by within-case analysis to understand how MLTCs affected each individual participant and their intervention engagement. JC created a database of individuals, including a summary of relevant codes for MLTCs including how they perceived their health, ability to change, locus of control, symptoms, goal progress and maintenance, and how these interacted. JC, RF, and TAR developed initial written themes with supporting evidence from the data. The wider team, including academic clinicians (GPs, clinical psychologists, community nurses, health psychologists), social care experts, and public contributors, reflected on these themes and quotes to incorporate a range of perspectives on the data. The themes were then refined through discussion and rewriting, to explore the specific experiences of those with MLTCs and engagement with health promotion interventions.

This study is reported in line with the Standards for Reporting Qualitative Research (SRQR) guideline^29^ (see supplementary table 1).

### Ethical approval and funding

The Social Care Research Ethics Committee (ref 20/IEC08/0013) gave a favourable ethical opinion and was funded by the National Institute for Health Research (NIHR) Health Technology Assessment (NIHR128334).

## Results

Of the 49 participants interviewed, the average age was 80.3 years, with a range of 66-94 years, with 65% female, 76% white British, and a mean Index of Multiple Deprivation level of 5.3 (range 1-10) based on their postal code (see Table 1). Participants lived with an average of 5.1 LTCs, with a range of 2-11, defined by combining participants medical notes (n=41) and self-reported LTCs (n=8). There were 32 unique health conditions reported, 17 of which occurred in more than 5 participants, including hypertension, chronic pain, diabetes, vision and hearing impairments, osteoporosis, thyroid disorders, asthma, and arthritis (see Table 2).

**Table 1.** Demographics of 49 participants living with MLTCs interviewed. Adapted from the HomeHealth process evaluation.^21^

Demographics	Frequency/Mean (SD)
**Age in years (at recruitment)**	80.3 (6.6)
**Gender**	
Female	32
Male	17
**Born in…**	
United Kingdom	34
Another country	15
**Ethnicity**	
White: English/Welsh/Scottish/Northern Irish/British	37
Any other White background	4
Black/African/Caribbean/Black British: African	2
Asian/Asian British: Indian	2
Irish	1
Any other ethnic group	3
**Sexuality**	
Heterosexual	46
Homosexual	1
Prefer not to say	2
**Highest level of education**	
No formal qualifications	19
General Certificate of Education/O levels or equivalent	11
Certificate of Secondary Education/A levels or equivalent	2
Higher national diploma or equivalent	3
Degree or higher	14
**Index of Multiple Deprivation (range 1-10)**^a^	5.3 (2.9)
**Number of LTCs**	5.1 (2.3)

^a^Higher deciles indicate less deprivation.

**Tables 2.** LTCs reported in at least 5 participants.

Long term condition	Number of participants	Percentage of participants
Hypertension	33	67%
Chronic painful disorders	27	55%
Diabetes	19	39%
Vision impairment	14	29%
Osteoporosis	13	27%
Thyroid disorders	13	27%
Hearing impairment	13	27%
Asthma	12	24%
Arthritis	12	24%
Coronary heart disease	11	22%
Depression	9	18%
Atrial fibrillation	8	16%
Chronic kidney disease	8	16%
Diverticulitis	7	14%
Stroke/TIA	5	10%
COPD	5	10%
Cancer	5	10%

We identified three main themes regarding the impact of MLTCs on engagement with and benefit from a behaviour change intervention and how to adapt interventions in the future: 1) Focus on symptoms rather than conditions, 2) Coping with and adapting to symptoms, and 3) Tailoring goal setting for MLTCs.

### Focus on symptoms rather than conditions

The impact of MLTCs on engagement varied greatly between individuals, however, this was dependent on the perceived burden of specific symptoms rather than presence of particular conditions or combination of conditions. Whilst having more diagnosed LTCs did make it more likely a person would say their symptoms had a bigger impact on their ability to engage with the intervention, some people with relatively few conditions (e.g., 2-4) felt the impact was *“quite bad actually”. (2128, Female, 87, 3 LTCs).* This was viewed to not link to specific sets of conditions, but rather the level of ongoing functional impairment it produced.

I mean I’ve had a heart attack but that doesn’t really bother me much. I’ve had cancer but that was only a mild one. Arthritis is just the worst one, along with sciatica and all the aches and pains that go with it. (3053, Female, 77, 8 LTCs)

The most reported area of functional impairment mentioned was mobility, and associated activities such as gardening, walking, cooking, socialising, and self-care, which were often aligned to the types of goals participants wanted to set. Pain was the symptom identified as mostly likely to impact daily activities and goal progress, which could be constant or fluctuate.

But I'm in constant pain and that is added to everything else I've got. So, the things I do and don't do, it depends on my pain level, what pain I'm in and things. (3036, Female, 69, 6 LTCs)

Another common symptom reported to affect mobility goals was incontinence. Some mentioned faecal incontinence, which led to: feeling unable to leave the house, having to plan ahead to know where toilets are, having to wear pads, carrying extra supplies when out, or having accidents. A few mentioned urinary incontinence and frequent urination, the latter of which was often attributed to diuretics, which participants felt curtailed their ability to completed planned behaviours until the side effect dissipated. Of those who mentioned wearing pads, all but one mentioned inconvenience due to irritation, 24-hour wear, or feeling dismissed that no other treatment was available.

I've been wearing pads for about 20 years, which is not nice, and it affects life, because these pads are 24/7 that you've got to wear and then the bowels started leaking as well. (3036, Female, 69, 6 LTCs)

Breathlessness, particularly when walking uphill, or dizziness also had a strong impact, making people feel unwell, needing to take breaks, and increasing their fear of trying new exercises due to the risk of falls.

If I go up a hill, I have to stop because I can’t get my breath, you know, but I can cope, I can manage, you know, but it’s not a nice feeling... when you can't breathe. (3071, Female, 83, 8 LTCs)

Fatigue was an important symptom for many; around half described fatigue as a limiting factor in daily activities (such as housework, shopping, and self-care), and was linked with a variety of LTCs, such as cancer, long COVID-19, thyroid disease, heart disease, depression, fibromyalgia, or insomnia. It was rarely considered as part of frailty, but rather part of getting older.

I can just about manage to lift the [shopping] bags into the car and then get them in here. And I usually sit down and fall asleep before I’ve got it all out of the bags. I’ve been falling asleep a lot lately. (3055, Female, 66, 3 LTCs)

Hearing impairment was common and perceived to affect socialising, especially in group settings, and shopping. A few participants had ceased socialising in groups due to the barrier of not being able to distinguish between many voices, despite wanting to set this as a goal. Visual impairment led to difficulty being dazzled in bright sunlight, feeling insecure, affecting outdoor activities, and reading.

Several participants highlighted low mood or anxiety as influencing their day-to-day life and goals. Reasons given included fear of falling, fear of catching COVID-19, complications of COVID-19, loneliness, and worries over a family member’s ill health/death. Participants reported low mood due to frustration at not being able to do what they wanted or had previously been able to do and could lower motivation to try within the intervention.

I’ve been active all my life actually. I used to do athletics, everything. Now it’s very demoralising. I don’t feel I can do anything. (2134, Female, 83, 11 LTCs)

Specific medications, when remembered, were mostly viewed as a means to an end for treating conditions considered adequately controlled or expected in later life, like heart disease, high blood pressure, diabetes, diverticular disease, or hyper- or hypothyroidism, and were not viewed particularly positively or negatively. However, some medications were singled out as problematic, such as sleeping pills, and painkillers, due to side effects such as fatigue. A few mentioned avoiding or decreasing medications due to side effects such as swelling with steroids or frequent urination with diuretics, or because they felt they did not need them.

I do have sleeping tablets prescribed by the doctor. I allow myself one a fortnight... I have to choose a day when I’m not going to be doing something early in the morning. (1112, Female, 87, 4 LTCs)

A particular challenge mentioned was fluctuations in symptoms, sometimes with a clear reason like pain medication wearing off or acute illness. Others said there was no clear reason for changes in their symptoms and viewed fluctuations simply as good and bad days. This group of participants reported difficulty in making plans around symptoms and instead planning activities on a day-to-day basis, which made sticking to an action plan difficult.

### Coping with and adapting to symptoms

There were many ways people reported coping with MLTC symptoms. The key difference in perceived impact appeared to be in how participants responded to the symptoms. Regardless of perceived symptom severity, many people said that purposefully keeping a positive outlook helped them cope with their difficulties and engage with the intervention, in comparison with others.

Those who felt that the impact of their MLTCs was low reported that life was going as well as could be expected for their age, that they try to forget about their health problems to just get on with it, not letting it stop them and minimising its impact on daily life. They seemed to have a strong internal locus of control.

I forget about it... Just carry on normal... If I get out of breath, I have a sit down and get my breath back. (2111, Male, 82, 5 LTCs)

Others were motivated by comparing themselves to other people they knew. Some viewed declines in health as a personal failing, blaming themselves for not achieving what they “should”. But for others, this could be positive and lead to determination to avoid a worse fate, and a strong desire to stay independent. They were aware of their need to change and used the decline of other people to motivate them to do this and engage with the goal setting process.

I had a cousin it was terrible, he got diabetes… since then he’s had his leg amputated. And I thought well, that’s not going to be me, you know. (3108, Male, 76, 6 LTCs)

Some participants struggled to cope with symptoms and mentioned that the impairment on daily life caused feelings of being depressed, demoralised, angry, or stressed. This was viewed in relation to feeling useless, unable to do things or like they had lost their identity. Even those with no diagnosis of depression or anxiety mentioned experiencing anxiety and low mood.

I just want to get on, but I can’t. And that’s where the, I don’t know, anger or the- Yes, because you just get fed up because you can’t do anything, you know. (1049, Female, 74, 6 LTCs)

Working with the support worker setting goals seemed to produce feelings of empowerment and self-confidence to combat these negative feelings and emotions. Participants acknowledged that without this support, they would have been worse than they currently were and could now have a more positive outlook on life.

I am an old lady, but I’m going to be one of these old ladies that shrivel up and don’t go out and- And I thought no, I’m not going to do that, I’ve got somebody who’s helping me and when my husband used to just say, “Leave it, leave it,” I can’t leave it any longer, I’ve got to do something for me. And you’ve- You- The team have given me so much confidence, help and I don’t know where I’d be without it because I think I’d have just gone further back. But now I look forward to each day. (3101, Female, 72, 2 LTCs)

### Tailoring goal setting for MLTCs

Some people chose to directly address and overcome MLTC-related barriers as part of the goal setting process. Those who engaged with goal setting often chose mobility-based goals to try and overcome the impairment arising from MLTCs. Goals improving balance, dealing with pain, reducing fear or risk of falling, or being able to do activities more easily were prioritised by these participants, with input from the support worker. These goals were either exercise-based or focussed on providing environmental aids or adaptations to overcome limitations.

So, I said that I would like to try and get my balance back... and [the support worker]- She went away and when she came next time, she brought all those exercises and it’s been- It’s really been good for me, you know. (3071, Female, 83, 8 LTCs)

I’m very scared about getting in and out the bath. Now I’ve got a rail, that was through [the support worker]. (1046, Female, 76, 5 LTCs)

Some discussed adapting their goals around the limitations of MLTC symptoms. This meant participants could choose to drop a goal or parts of a goal completely if symptoms inhibited the ability to achieve it, either temporarily if symptoms were acute, or permanently when symptoms were ongoing (for example, removing exercises that caused more pain from the plan). This was reported as reasonable and acceptable.

It depends on your health actually. When one is 100% okay, last week I had a cough, coughing most of the time and I didn’t feel like eating anything. This kind of thing. So, during that period I didn’t do many exercises because first of all I was weak in the sense that I wasn’t eating much, I was coughing and aching all over the place. These periods come and go which is not unexpected at my age. (2021, Male, 85, 5 LTCs)

Others ignored their symptoms and persisted with goals, reasoning that they may as well try their best to achieve what they could as the symptoms would continue regardless. An example is, even when people had problems with mobility, they still reported determination to walk when they could.

I don’t walk anywhere near as well as I used to, I still try to but I’ve got rheumatoid arthritis as well which was about a year ago which doesn’t help but I try and ignore it and just do what I can do. (2010, Male, 77, 5 LTCs)

Those who actively planned around the impact of symptoms had usually already found suitable activities and adaptations through a process of trial and error, such as planning new activities for certain times of day when symptoms or medication side effects were lower, using aids such as continence pads, or accessing social support.

I had problems with my bowel and my bladder, and they’ve happened in the street shopping… I’ll do anything that I did 10, 20 years ago if I can, but now I’ve got to be careful if I go out on the bus. I have to take stuff with me, but I still go. I just take Wet Wipes and a pair of spare pants and things, and take an Imodium before I go, you know. (2095, Female, 81, 3 LTCs)

Some people did not want to engage in goal setting, as they felt they could not make substantial changes or did not want to change. However, they still found value in brief and low-level support to identify any key resources, aids, or simple actions they could undertake to maintain their health.

People that you point to that you trust as well so if it comes within your recommendation, it’s because you know them and you have some faith in their decency and honesty and trustworthiness. (1019, Male, 77, 7 LTCs)

To help participants get their symptoms under control, to enable participants to engage fully in the behavioural goal they had set, the support workers often acted as care coordinators, supporting participants to access healthcare services, like their GPs or mobility aids. Accessing these services post-pandemic was a particular challenge, delaying treatment and potentially causing participants’ LTCs and associated symptoms to deteriorate at a faster rate.

[The support worker] was concerned about not being able to see a doctor. That was her biggest concern… That’s my biggest concern is seeing a doctor. I went down with a chest infection just after Christmas. Managed to get through and speak to a doctor. He said you need antibiotics. It took three days to get them... The other problem is… I’ve had a chest x-ray and the scan. Now that’s over a month ago. And I’ve had nothing back from the doctors to say yes, nay, or no. And that’s what I’m finding more aggravating. And this is what [the support worker] was trying to sort out for me. (3108, Male, 76, 6 LTCs)

In addition to care coordination, participants spoke about how the support worker gave them the confidence to advocate for their own health and “*insist*” they received the help they needed from their GP and other healthcare professionals, which could help them to achieve health related goals in the future.

I think talking to [the support worker] - You know, she just sat and listened to me, but it gave me the confidence to do these things, so yes in that way it helped a lot. That’s I would say a bit plus. It gave me confidence. (1049, Female, 74, 6 LTCs)

## Discussion

Through interviews with people living with MLTCs and mild frailty who had received a personalised health promotion intervention, which aimed to help older adults with mild frailty maintain their independence, we explored the unique barriers and facilitators to engagement with the intervention and the impacts on their approach to goal setting.

One key finding was that the impact of MLTCs mainly related to their symptom burden upon participants, rather than particular types or patterns of LTCs. This in turn was linked with the level of functional impairment symptoms generated, particularly around mobility, and common problematic symptoms were pain, incontinence, breathlessness, fatigue, hearing impairment, low mood, and anxiety, relating to their physical capability. Whether or not people reported feeling able to manage, despite their troublesome symptoms, often depended on how they positioned or perceived themselves in relation to other people, related to their psychological capability. Those who felt they were doing well, in comparison to others of their age or with similar symptoms and/or conditions, had a more positive outlook and were motivated to keep going. However, those who felt that they were doing worse than others of similar age or impairment felt that they ‘should’ be doing more to help improve their symptoms and wellbeing, but were rarely doing this, leading to negative emotions and feelings, such as shame, guilt, and low mood. Focussing on MLTC symptom management worked as a good strategy to engage people in the goal setting process. Permanently or temporarily adjusting goals based on people’s abilities and the fluctuation in their symptoms associated with their LTCs was essential for some, whereas others wanted to push through and continue despite the challenges their conditions presented. Therefore, focussing on the effort of working towards goals to change behaviour rather than on goal progress itself is likely to be more beneficial and motivating for people living with MLTCs who manage the associated symptoms daily.

### Results in context

Our findings are consistent with previous work highlighting the challenges older adults face when engaging with goal setting.^30,31^ In the context of frailty and MLTCs, we have found that managing the symptoms of the conditions, such as pain, incontinence, and difficulty with mobility, is more important to people living with MLTCs than focussing on the conditions themselves. This highlights a need to shift the guidance for the management of people living with MLTCs, with a more holistic, functional, symptoms focussed approach, rather than the fragmented condition specific approach often delivered in clinical practice.^31,32^ Guidelines need to be replaced or updated to look at the person as a whole and help to treat and/or improve the most impactful symptoms the person experiences and adapt the focus depending on fluctuations in symptom severity.^33^ This approach will help people with frailty and MLTCs to overcome the complexity of health systems in which different LTCs are currently managed, through improved care coordination, a role which the support workers often fulfilled in the HomeHealth trial.^22,34^

In the context of MLTCs and mild frailty, people who are frail are more likely to have health conditions which impair physiological reserve, such as COPD and diabetes, which are also more likely to have fluctuating symptoms.^35^ Whilst 72% of people with frailty are living with MLTCs, only 16% of people with MLTCs are considered frail.^36^ The findings here may be more applicable to those with frailty than those with MLTCs. The combination of MLTCs and frailty worsens health outcomes, in comparison to either group alone, including higher rates of hospital admissions, longer stays in hospital, and higher mortality rates.^35,36^ Therefore, identifying and implementing ways to support this population to engage in behaviour change and goal setting to prevent decline is important.

Complex interventions, like the HomeHealth intervention tested in this clinical trial, which include goal setting and care coordination are self-reported as relevant and helpful by people with MLTCs, even though not found to be statistically effective.^37^ However, for those who feel they cannot make substantial changes or do not want to change, brief and low-level support to identify any key resources, aids, or simple actions they can undertake to maintain their health may be most useful. For those motivated to engage in goal setting behaviours, more complex goal setting interventions are likely to be more helpful and may need to focus on ensuring goals are realistic and achievable, or planning how to adapt behaviour changes around symptom fluctuations based on strategies people already use. In two recent systematic reviews, behaviour focussed goal setting and outcome focussed progress monitoring were found to be linked with improved outcomes in people with MLTCs, as well as problem solving, action planning and social support.^18,38^ Our work shows that positive feedback on engaging with behaviour change and goal setting is important for those living with MLTCs who have an external locus of control. However, it is important to acknowledge that fluctuations in symptoms, linked to MLTCs, may make behaviour change difficult, highlighting the need for adaptation, problem solving, and positive feedback on progress.^18,38^ Whilst these reviews highlight that monitoring outcomes improves effectiveness; our findings suggest that people with MLTCs are more engaged when emphasis is placed on effort for engagement with behaviour change rather than goal progress. This may be because it supports people to remain engaged when they experience health-related setbacks.

Many participants with MLTCs were able to engage with the intervention and make progress with their goals throughout the intervention period. Participants self-reported that they felt they were still maintaining the progress they had made throughout the intervention, but difficulties arose when they constantly had to adapt their goals and behaviour around fluctuating symptoms and physical capability. This can make both goal setting and maintenance of goal progress even harder in this population, linking in with the literature for people with single LTCs having difficulty maintaining progress post-intervention.^39^ Therefore, behaviour change interventions for people with MLTCs need to have a stronger focus on how behaviours can be maintained when external support is removed and declines in health occur.

### Strengths and limitations

This research was embedded within a process evaluation of a randomised controlled trial, with an experienced research team using rigorous research methodology. We aimed to recruit a diverse sample of participants, researchers, and public contributors, to ensure the views of people with different combinations of LTCs were included (both physical and mental). However, we were limited to sampling from those who received the HomeHealth intervention, who were a specific sample of those living with mild frailty. Therefore, some concepts may not be transferable to those without mild frailty. The multidisciplinary team have a range of lived with and clinical experiences of MLTCs, which were discussed in team meetings to ensure a range of perspectives were taken into account without overly influencing the interpretations.

### Future research and implications

These results highlight the need to design and deliver behaviour change interventions for people with MLTCs with a symptom-centred approach, identifying those which are generating the most impact on living well, and to find ways to target different subsets of people with MLTCs by their symptom burden. Our work suggested that interventions for those who do not think they can change could include low-level brief support, with positive reinforcement of effort towards behaviour change rather than the behaviour itself. Whereas those motivated to change are likely to engage with more complex support that focuses on managing the most impactful MLTC symptoms and building on existing strategies people use to overcome them to help people to achieve their goals around their symptoms. Further work also needs to find ways to support older adults with MLTCs to manage and plan for the fluctuation in their symptoms to ensure continued engagement with behaviour change and improved outcomes over time.

## Conclusion

People living with MLTCs do engage with health promoting behaviour change interventions targeted at maintaining their independence. Emphasis on the effort for behaviour change rather than goal progress, may support motivation in people who experience health-related setbacks to help prevent a decline. When attempting behaviour change, it is important to help people to manage the problematic symptoms related to their LTCs and the treatment they take for them e.g., pain, incontinence, breathlessness, and fatigue, to ensure they have new strategies and can build on existing ones to continue to engage when barriers from their symptoms arise.

## Supplemental material

Supplemental material - The impact of multiple long-term conditions on engaging with and maintaining behaviour change in older people with mild frailty: A qualitative study Supplemental material for The impact of multiple long-term conditions on engaging with and maintaining behaviour change in older people with mild frailty: A qualitative study by Tasmin Rookes, Jessica Catchpole, Kate Walters, Yolanda Barrado-Martín, Sarah Kalwarowsky, Christina Avgerinou, Benjamin Gardner, Rebecca L. Gould, Paul Chadwick, Jane Hopkins, Vari M. Drennan, Kalpa Kharicha, Louise Marston, Claire Jowett, Rashmi Kumar, Rekha Elaswarapu and Rachael Frost in Journal of Multimorbidity and Comorbidity.

## Data Availability

The data that support the findings of this study are available from the corresponding author upon reasonable request.*

## ORCID iDs

Tasmin Alanna Rookes https://orcid.org/0000-0001-6330-7059

Jessica Catchpole https://orcid.org/0000-0001-7901-1797

Kate Walters https://orcid.org/0000-0003-2173-2430

Yolanda Barrado-Martín https://orcid.org/0000-0002-9912-6345

Sarah Kalwarowsky https://orcid.org/0009-0009-3817-0244

Christina Avgerinou https://orcid.org/0000-0002-1445-1676

Benjamin Gardner https://orcid.org/0000-0003-1223-5934

Rebecca L. Gould https://orcid.org/0000-0001-9283-1626

Vari M. Drennan https://orcid.org/0000-0002-8915-5185

Kalpa Kharicha https://orcid.org/0000-0002-2975-2084

Louise Marston https://orcid.org/0000-0002-9973-1131

Rachael Frost https://orcid.org/0000-0003-3523-0052
